# A Blend Consisting of Agaran from Seaweed *Gracilaria birdiae* and Chromium Picolinate Is a Better Antioxidant Agent than These Two Compounds Alone

**DOI:** 10.3390/md21070388

**Published:** 2023-06-29

**Authors:** Yara Campanelli-Morais, Cynthia Haynara Ferreira Silva, Marina Rocha do Nascimento Dantas, Diego Araujo Sabry, Guilherme Lanzi Sassaki, Susana Margarida Gomes Moreira, Hugo Alexandre Oliveira Rocha

**Affiliations:** 1 Programa de Pós-Graduação em Bioquimica e Biologia Molecular, Universidade Federal do Rio Grande do Norte (UFRN), Natal 59078-900, Brazil; campanelliy@gmail.com (Y.C.-M.); cynthiahaynara@gmail.com (C.H.F.S.); marina.dantas.111@ufrn.edu.br (M.R.d.N.D.); susana.moreira@ufrn.br (S.M.G.M.); 2 Dapartamento de Bioquímica, Universidade Federal do Rio Grande do Norte (UFRN), Natal 59078-900, Brazil; popoh.diego@gmail.com; 3 Departamento de Bioquímica e Biologia Molecular, Setor de Ciências Biológicas, Universidade Federal do Paraná (UFPR), Curitiba 81531-980, Brazil; sassaki@ufpr.br; 4 Departamento de Biologia Celular e Genética, Universidade Federal do Rio Grande do Norte (UFRN), Natal 59078-900, Brazil

**Keywords:** nutraceutic, oxidative stress, Rhodophyta, sulfated polysaccharide

## Abstract

A blend refers to the combination of two or more components to achieve properties that are superior to those found in the individual products used for their production. *Gracilaria birdiae* agaran (SPGb) and chromium picolinate (ChrPic) are both antioxidant agents. However, there is no documentation of blends that incorporate agarans and ChrPic. Hence, the objective of this study was to generate blends containing SPGb and ChrPic that exhibit enhanced antioxidant activity compared to SPGb or ChrPic alone. ChrPic was commercially acquired, while SPGb was extracted from the seaweed. Five blends (B1; B2; B3; B4; B5) were produced, and tests indicated B5 as the best antioxidant blend. B5 was not cytotoxic or genotoxic. H_2_O_2_ (0.6 mM) induced toxicity in fibroblasts (3T3), and this effect was abolished by B5 (0.05 mg·mL^−1^); neither ChrPic nor SPGb showed this effect. The cells also showed no signs of toxicity when exposed to H_2_O_2_ after being incubated with B5 and ChrPic for 24 h. In another experiment, cells were incubated with H_2_O_2_ and later exposed to SPGb, ChrPic, or B5. Again, SPGb was not effective, while cells exposed to ChrPic and B5 reduced MTT by 100%. The data demonstrated that B5 has activity superior to SPGb and ChrPic and points to B5 as a product to be used in future in vivo tests to confirm its antioxidant action. It may also be indicated as a possible nutraceutical agent.

## 1. Introduction

Algae, in addition to their extreme ecological importance (providing about 70 to 90% of the planet’s O_2_), also have value in the food industry. Around 178 million tons of food is obtained annually from the aquatic environment, of which 36 million tons is composed of macroalgae [1]. These algae have high nutritional value and low caloric content [2]. They are rich in carbohydrates (polysaccharides that mostly act as fibers), proteins (containing all essential amino acids), and long-chain polyunsaturated fatty acids. Additionally, they contain significant amounts of vitamins, minerals (with values about ten times higher than those found in terrestrial plants), and biologically active secondary metabolites, mainly polyphenols, some of which are exclusive to seaweeds [3].

Examples of the main cultivated macroalgae include nori (*Pyropia yezoensis*), dulse (*Palmaria palmata*), wakame (*Undaria pinnatifida*), Irish moss (*Chondrus crispus*), *Himanthalia elongata*, and seaweeds of the genera *Gelidium*, *Gracilaria*, and *Pterocladia* [4]. In Brazil, seaweed production is low, and on the northeast coast of the country (~3500 km), which is characterized by a tropical climate, the only cultivated seaweed is *Gracilaria birdiae* [5].

*G. birdiae* is cultivated for the extraction of agar-agar [6], a hydrocolloid formed by the heterogeneous mixture of two polysaccharides: agarose (the gelling component) and agaropectin (which has a low ability to form a gel). However, this seaweed also synthesizes another polysaccharide, a sulfated agaran [7]. This agaran contains a predominant disaccharide unit formed by 3-substituted β-D-galactose, linked to position 4 of an α-L-galactose, and some of these residues are sulfated at C6. The latter may be, in part, in the form of 3,6-anhydro-α-L-galactose, and residues of other monosaccharides, such as β-D-xylose, can also be found [7]. The *G. birdiae* agaran has already been described as anti-inflammatory [8], as a protector against tissue damage caused by naproxen [9] and trini-trobenzenesulfonic acid [10], as an anticoagulant [11], as an agent that prevents pre-adipocyte adipogenesis under culture conditions [12], and as an antioxidant agent [13].

Agarans from other seaweeds have also been characterized as pharmacologically active agents. Agarans from *G. debilis* have antioxidant activity and exhibit blood clotting prevention; as an example, heparin is an anticoagulant used in clinical medicine [14]. Agarana from the seaweed *G. opuntia* has been identified as a potential compound for treating hyperglycemia in diabetic patients due to its ability to inhibit α-amylase and α-glucosidase [15]. Agarans are also recommended as antidiarrheal agents in cases of cholera toxin contamination. It has been proposed that agarans compete with cholera toxin for the GM1 receptor, thereby blocking the toxin’s adhesion to small intestinal epithelial cells [16]. Agarans can also be considered immunomodulators, acting as probiotics [17,18] and exhibiting antibacterial activity against human pathogens such as *Bacillus subtilis*, *Enterobacter aerogens*, *Escherichia coli*, *Klebsiella oxytoca*, *Pseudomonas aeruginosa*, *Salmonella typhi*, *Salmonella paratyphi*, *Salmonella choleraesuis*, *Staphylococcus aureus*, and *Vibrio cholerae* [19,20]. However, the specific mechanisms of action against these microorganisms still need to be elucidated. These findings demonstrate the significant potential of agarans as bioactive molecules and highlight the need for further studies to uncover more information about these polysaccharides.

The definition of ‘blend’ is somewhat general, and it involves the objective of mixing two or more components to obtain properties and/or applicability superior to those found in the products used for their production [21]. Blends have gained significant attention due to their wide range of applications in various fields, including food and pharmaceutical industries [22,23,24]. The incorporation of different compounds into blends offers numerous benefits such as improved functionality, enhanced bioavailability, and synergistic effects. These properties make blends highly versatile and attractive for developing innovative products with enhanced performance and health benefits [25,26,27,28,29,30,31].

In the food industry, blends offer an opportunity to create new flavors, textures, and nutritional profiles. By combining different ingredients, such as natural extracts, spices, or functional additives, food blends can provide unique sensory experiences and address specific dietary needs. Additionally, blends can improve the stability and shelf life of food products, ensuring their quality and safety throughout the distribution chain [31,32,33].

In the pharmaceutical field, blends play a crucial role in formulating drug delivery systems [34]. By incorporating active pharmaceutical ingredients (APIs) into blends, the controlled release of drugs can be achieved, resulting in optimized therapeutic effects, reduced side effects, and improved patient compliance. Moreover, the combination of APIs with excipients or carriers in blends can enhance drug solubility, bioavailability, and overall efficacy [35,36,37].

Furthermore, blends offer opportunities for synergistic effects, where the combined action of different compounds results in enhanced functional properties or physiological responses. This synergism can lead to improved antioxidant activity, antimicrobial efficacy, or even targeted drug delivery, opening new possibilities for developing advanced formulations in various industries [38,39,40].

In this context, many polysaccharides and other low-toxicity polymers are used in the production of blends, such as hyaluronic acid [22], starch [23,25], dextrans [24], polylactic acid [26], carrageenans [24], polyethylene glycol [29], alginates [30], and chitosans [31]. However, no papers were found that demonstrate the production of blends containing agarans.

Chromium picolinate (ChrPic) is a complex formed by the coordination of picolinic acid with chromium III (Cr^3+^). It is an organic compound in which three picolinic acid residues are attached to a chromium atom [41]. ChrPic ingestion has been shown to improve mood [42], appetite [43], and glucose regulation in several groups of psychiatric patients [44,45]. ChrPic may also be useful in the treatment of binge eating disorder (BED) [46], depression [47] and premenstrual dysphoric disorder (PMDD) [48]. Studies also suggest that chromium inhibits the hepatic enzyme hydroxymethylglutaryl-CoA reductase [49], which decreases plasma cholesterol levels and helps with weight loss [50]. Dietary supplementation with brown seaweed and chromium picolinate has been shown to have an anti-inflammatory action [51]. In addition, ChrPic has also been shown to be an antioxidant agent in vivo, improving several antioxidant parameters such as catalase, glutathione reductases, glutathione peroxidase, and superoxide dismutase in animals supplemented with ChrPic [52,53].

Oxidative stress is a condition characterized by an imbalance between the production of reactive species and the body’s ability to detoxify these reactive intermediates, resulting in cellular damage [54]. The detrimental effects of oxidative stress have been associated with various diseases, including neurodegenerative disorders, cardiovascular diseases, cancer, Alzheimer’s disease, and inflammatory conditions [55]. Antioxidants are compounds that possess the ability to counteract harmful reactive species, which can otherwise inflict damage on cells and contribute to the development of the chronic ailments mentioned earlier [56].

An ideal antioxidant should possess certain characteristics. Firstly, it should be safe for human and animal consumption, ensuring that its use does not pose any health risks. Secondly, it should exhibit bioavailability, meaning that it can be effectively absorbed and utilized by the body. Thirdly, it should have the ability to function in diverse environments, including both aqueous and lipid-based environments, and in different pH levels. Moreover, it should demonstrate the capacity to efficiently neutralize a wide range of reactive species. It is important that the antioxidant is resistant to chemical degradation, allowing it to maintain its antioxidant activity over an extended period. Lastly, an ideal antioxidant should be cost-effective to produce, ensuring accessibility to a broad population [57].

It is worth noting that no single antioxidant compound has all these characteristics. Therefore, products composed of more than one type of antioxidant would have a more beneficial effect than those consisting of only one antioxidant compound. In this context, the main objective was to produce different blends consisting of red seaweed agaran, which is a natural polymer obtained from a renewable source, and ChrPic. Both are known as antioxidant agents and possess other properties. It is expected that the produced blends combine the action of these two compounds and, therefore, exhibit superior antioxidant activity compared to the agents from which they originated.

## 2. Results and Discussion

### 2.1. Extraction and Characterization of SPGb

The procedures described in the Methods section led to the obtainment of 17.98 g of SPGb, which corresponds to 7.74% SPGb per 100 g of dry seaweed. This yield was higher than the 1.3% obtained by Medeiros et al. [58]. The difference in yield can be attributed to the use of a basic medium and the ultrasound technique associated with proteolysis in this work, which were not used by Medeiros et al. [58]. According to Fidelis et al. [11], the presence of a base in the medium promotes the hydrolysis of macroscopic structures and erodes the surface of the tissue, allowing for greater access to shock waves to previously protected sites. This promotes greater extraction of polysaccharides. Ultrasound causes the formation of microbubbles due to the action of mechanical sound energy, and these microbubbles grow and collapse, releasing energy capable of breaking existing chemical bonds between molecules found in the tissue. This creates new sites for SPGb extraction. The use of ultrasound/base also enhances the action of enzymes by increasing the contact surface area for them to act. Proteolytic enzymes can degrade proteins in the extracellular matrix of the seaweed, promoting a greater release of SPGb, which is typically trapped in the matrix because it is associated with its proteins [59].

Although the influence of base concentration on the efficiency of the extraction process was not evaluated in this work, other studies have demonstrated that the use of NaOH can increase the extraction of polysaccharides. However, the use of a concentration above 1.8 M decreases the efficiency of the process, likely due to the hydrolysis of the extracted polysaccharides [60]. This indicates that there is a limit to the amount of base that can be used in the procedure.

Table 1 summarizes the SPGb chemical analysis results. Proteins are common contaminants in sulfated polysaccharide solutions, and their amount depends on both the species of seaweed used as a source of polysaccharide and the extraction method employed [61]. The SPGb extraction method proposed by Maciel et al. [7] is easy to reproduce and inexpensive; however, it yields SPGb with about 8% protein contamination. To reduce this contamination, Fidelis et al. [11] proposed the addition of a proteolysis step to the SPGb extraction method. In this work, proteolysis was added to the extraction method, resulting in lower protein levels in the obtained SPGb compared to those described by Maciel et al. [7]. Furthermore, the protein levels observed here were like those reported in studies that also used proteolysis during the SPGb extraction process [13,21].

Phenolic compounds were also evaluated, but they were not detected in SPGb. This is an important finding because the absence of phenolic compounds may rule them out as the responsible molecules for the antioxidant activity that SPGb may have.

The sulfate content in SPGb, which was around 10%, can be considered high compared to other sulfated polysaccharides from algae of the genus *Gracilaria*, as they are usually less sulfated than polysaccharides from other seaweeds. The value found in this study was higher than that described by Maciel et al. [7], who reported a value of around 6%. In any case, the main monosaccharides identified in SPGb were galactose, glucose, arabinose, and xylose (Table 1). The monosaccharide composition found in SPGb was not similar to that described previously [7]; these authors did not find glucose, arabinose, and xylose. The difference could be explained by the fact that the seaweeds were collected in different years, leading to changes in the structure of the polysaccharide, including different monosaccharide composition as shown by Rioux et al. [62]. The authors demonstrated that the sulfate content and monosaccharide composition of a sulfated galactofucan from the seaweed *Saccharina longicruris* changed over the course of different months in which the seaweed was collected (May, August, and November 2005, and June 2006).

In summary, the absence of phenolic compounds in SPGb may exclude them as the responsible molecules for its antioxidant activity, while the high sulfate content in SPGb, which is higher than that found in other *Gracilaria* species, may contribute to its biological activities, such as antioxidant activity, due to the known critical role of sulfate groups in the biological activity of sulfated polysaccharides.

**Figure 1:**
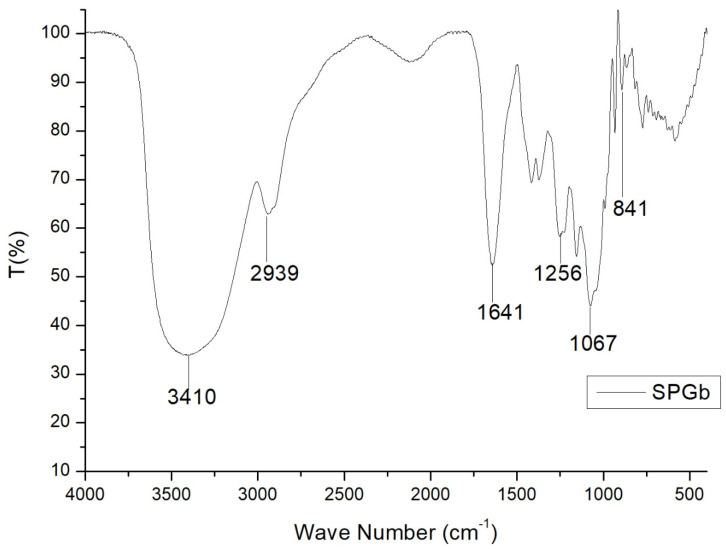
Infrared spectra of SPGb in the 4000 to 500 cm^−1^ regions.

Infrared spectroscopy (FT-IR) is a valuable and commonly employed analytical technique for investigating the physicochemical characteristics and conformational properties of substances. With this in mind, we utilized FT-IR to examine SPGb, and the resulting spectra can be observed in Figure 1.

Bands in both spectra with absorption close to the 3410 cm^−1^ and 2939 cm^−1^ regions were attributed to the O-H stretching vibration of O–H and C–H, typical of monosaccharides [63,64]. The characteristic absorptions of sulfate were identified in the FT-IR spectra of SPGb: band around 1256 cm^−1^ for an asymmetric S=O stretching vibration and band around 1067 cm^−1^ for a symmetric C–O vibration associated with a C–O–SO_3_ group. The band at 841 cm^−1^ was caused by the bending vibration of C–O–S [11].

**Figure 2:**
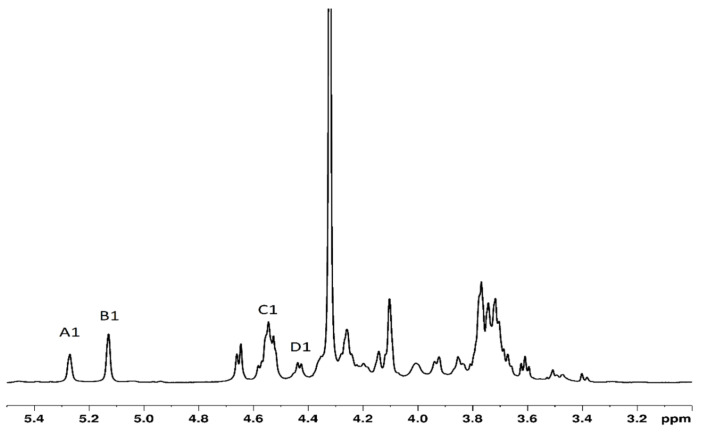
^1^H^1^D spectrum of SPGb with the anomeric hydrogens assigned.

The identity of SPGb was confirmed by the NMR spectrum shown in Figure 2, which highlights the signals of the anomeric hydrogens of the sugar residues present in SPGb. The identified signals of the assigned anomeric hydrogens were as follows: A1—5.30 ppm (α-L-galactose-6 sulfate (L-6S)); B1—5.17 ppm (3,6 α-L-anhydrogalactose); C1—4.57 ppm (β-D-galactose); and D1—4.48 ppm (β-D-galactose linked to L-6S units).

**Figure 3:**
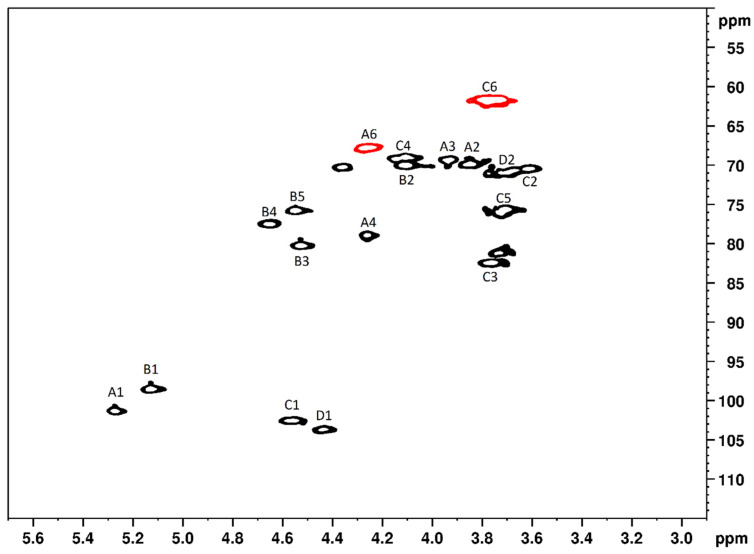
HSQC spectrum of SPGb labeled. The letters A–D indicate the 4 spin systems. The numbers indicate the correlation sign of the signed carbon and hydrogen pair. For example, A1 indicates a correlation sign between carbon 1 and hydrogen 1 of system A. The correlation signals between H6 and C6 are highlighted in red.

The signals of the other atoms found in the disaccharide units of SPGb were assigned with the help of two-dimensional NMR. Thus, four spin systems were detected, indicating the presence of four monosaccharides in the SPGb composition. Figure 3 presents a SPGb HMQC spectrum where these four spin systems are marked, which correspond to four different monosaccharides: A: α-L-galactose-6 sulfate (L-6S); B: 3,6 α-L-anhydrogalactose; C1: β-D-galactose; and D: β-D-galactose liked to L-6S unit.

The units consist of β-D-galactose residues, and their respective chemical shifts were determined by NMR analysis (Table 2). The values are very similar to those described by Maciel et al. [7] and confirm the identity of PSGb as an agaran.

### 2.2. Antioxidant Activity of SPGb and ChrPic

The ChrPic was commercially acquired. Therefore, its characterization was not necessary. Thus, ChrPic and PSGb were evaluated as antioxidant agents using five methods: total antioxidant capacity (TAC); hydroxyl radical scavenging; superoxide radical scavenging; iron chelation; and copper chelation.

Regarding the TAC test, an activity of 87 mg ascorbic acid equivalents per gram of sample was identified for SPGb, while ChrPic yielded 25 mg ascorbic acid equivalents per gram of ChrPic.

**Figure 4:**
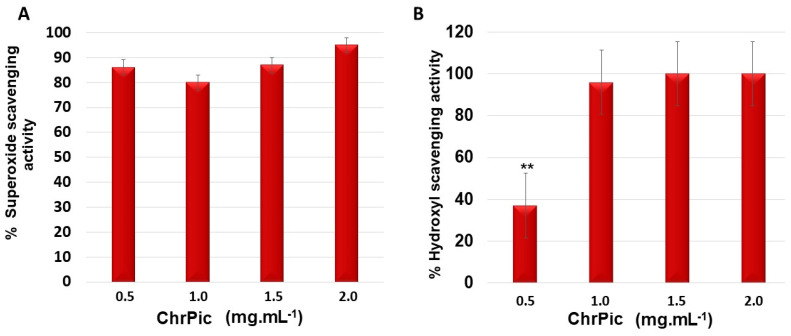
Radical scavenging activity of ChrPic. (**A**) Superoxide scavenging activity. (**B**) Hydroxyl scavenging activity. ** ChrPic 0.5 mg·mL^−1^ vs. ChrPic 1.0 mg·mL^−1^ (*p* < 0.05). Three independent assays were performed in triplicate, and data were analyzed using ANOVA and Tukey’s test.

In the tests for scavenging of superoxide and hydroxyl radicals, no activity was detected with SPGb (at concentrations from 0.1 to 2.0 mg·mL^−1^), which had already been described by other authors [13,14]. On the other hand, as can be seen in Figure 4, an activity of about 80% was identified for ChrPic at all evaluated concentrations. In the hydroxyl radical scavenging test, an activity of about 100% was identified at a concentration of 1.0 mg·mL^−1^, which did not change with the increase in the evaluated concentration (Figure 4B).

**Figure 5:**
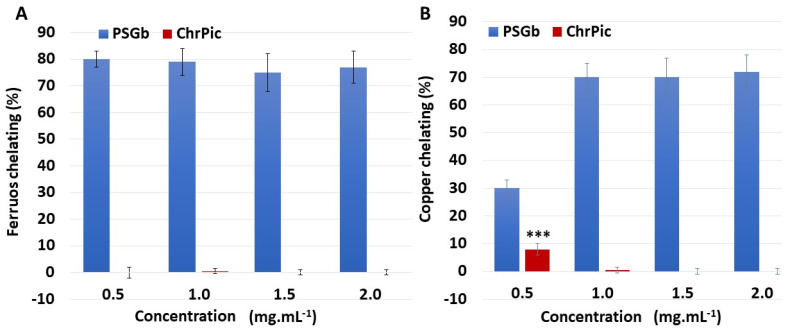
PSGb and ChrPic chelating effect on ferrous and copper ions. (**A**) Ferrous chelating activity. (**B**) Copper chelating activity. Data are expressed as mean ± standard deviation. *** ChrPic 0.5 mg·mL^−1^ vs. ChrPic 1.0 mg·mL^−1^ (*p* < 0.001). Three independent assays were performed in triplicate, and data were analyzed using ANOVA and Tukey’s test.

Regarding iron chelation, no activity was identified for ChrPic (Figure 5A) at any of the concentrations tested (ranging from 0.5 to 2.0 mg·mL^−1^). On the other hand, PSGb (Figure 5A) demonstrated high activity (~80%) even at the lowest concentration evaluated. However, this activity did not change with increasing concentration.

ChrPic also did not show any copper chelating activity, except when it was evaluated at a concentration of 0.5 mg·mL^−1^. In this case, ChiPic exhibited copper chelating activity, but significantly lower than that observed with SPGb (Figure 5B). In contrast, PSGBb (0.5 × 5 mg·mL^−1^) showed an activity of around 30%, which increased to around 80% when evaluated at higher concentrations of SPGb (Figure 5B).

The metal chelation data obtained here are similar to those described for SPGb by other authors [11,13] and indirectly demonstrate that the polysaccharide obtained here is similar to those described and characterized by these authors.

No studies were found in which the antioxidant activity of ChrPic was evaluated, which also made it impossible to compare the data obtained here with those from the literature.

The fact that ChrPic presented antioxidant activity in tests where SPGb did not show activity was something that drove the production of blends, as it was initially imagined that there could be complementarity between the activities of SPGb and ChrPic.

### 2.3. Antioxidant Activity of Blends

The data obtained from the antioxidant tests demonstrated that the two compounds evaluated here have the potential to complement antioxidant agents. Thus, to evaluate this hypothesis, five blends were developed with different concentrations of their components, as described in the methods section, and they were named B1, B2, B3, B4 and B5. These five blends were then evaluated using the same tests that were performed on SPGb and ChrPic. It is worth noting that, to obtain a better comparison between the blends, they were evaluated at only one concentration, 1.0 mg·mL^−1^, which was the concentration at which maximum activity was obtained with SPGb and ChrPic in the different tests.

**Figure 6:**
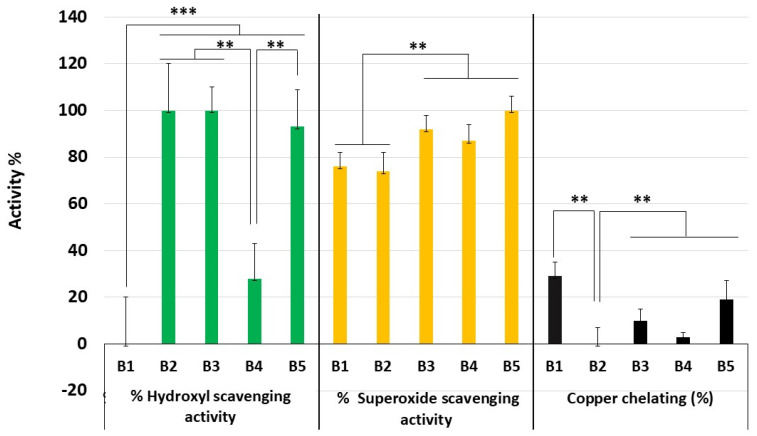
Antioxidant activity of the blends determined by three different tests. All blends were evaluated at a concentration of 1.0 mg·mL^−1^. Data are expressed as mean ± standard deviation. Three independent assays were performed in triplicate, and data were analyzed using ANOVA and Tukey’s test. *** *p* < 0.001; ** *p* < 0.05.

The data obtained from the hydroxyl scavenging test are shown in Figure 6. With the B1 blend, very low activity was found, with an average value of zero. With B4, a low activity of around 30% was also obtained. The highlight was for blends B2, B3, and B5. In these tests, these blends obtained about 100% activity at the evaluated concentration.

In Figure 6, the data obtained from the superoxide radical scavenging test are also compiled. Once again, the effect of the different blends was not similar. However, all blends showed activity above 70% scavenging. In fact, two groups are observed: one formed by B1 and B2, which were less effective and showed activity around 75%. The other blends form the second group, with an emphasis on B5, which was the most effective in this second group. For the chelation analyses, all five blends showed results below 30% of the activity with copper (Figure 6) and no chelating activity with iron.

After preparing the blends, they were tested for their antioxidant activity. It was observed that the presence of SPGb in the blends did not give them a superior metal chelating activity compared to that of SPGb alone in the in vitro tests. Even B1, which showed copper chelating activity of around 29%, respectively, had an activity about 60–70% lower than that of SPGb. It is possible that the reason for not obtaining the chelating activity of metals in the blends is because ChrPic contains a metal, in this case chromium, which could interact with copper and iron binding sites present in SPGb, thus reducing the ability of the blend to chelate copper and iron compared to SPGb alone (Figure 5).

Overall, in the tests where the blends showed pronounced activity, it was observed that there was a difference in activity between the different blends. Both B3 and B5 excelled in these tests. Since B5 had almost twice the copper chelating activity of B3, it was chosen for further testing.

### 2.4. Evaluation of the Cytotoxic and Genotoxic Effect of B5

The next step was to evaluate whether B5 exhibited cytotoxicity. For this purpose, 3T3 cells and CHO-k1 cells were chosen, as described in the Methods section. In this case, SPGb and ChrPic also had their cytotoxic potential evaluated.

**Figure 7:**
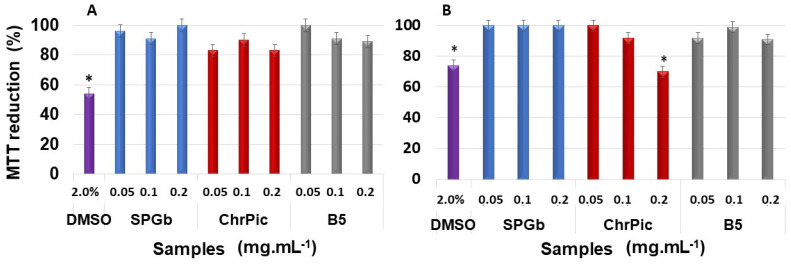
The 3-(4,5-dimethylthiazol-2-yl)-2,5-diphenyl-tetrazolium bromide colorimetric (MTT)-reducing activity of fibroblast murine 3T3 cells (**A**) or Chinese ovarian Hamster (CHO-K1) cells (**B**) treated with SPGb, ChrPic or B5 24 h. Data are expressed as mean ± standard deviation. The cells that were not exposed to any of the compounds were considered as the negative control, and the MTT reduction value of these cells corresponds to 100% MTT reduction. * Sample versus negative control (*p* < 0.05). Three independent assays were performed in triplicate, and data were analyzed using ANOVA and Tukey’s test.

Regarding 3T3 cells, it can be seen in Figure 7B that when exposed to the three compounds, these cells’ ability to reduce MTT was reduced by a maximum of 17% in the case of exposure to ChrPic (0.02 and 0.5 mg·mL^−1^). However, there was no significant difference compared to the control, the same occurring with the other samples (SPGb and B5).

With CHO-k1 cells, the toxicity results (Figure 7B) were similar to those observed with 3T3 cells. That is, signs of cytotoxicity were only found with ChrPic at a concentration of 0.2 mg·mL^−1^. However, this effect was not transferred to B5, as no signs of cytotoxicity were found with this compound in any of the evaluated cell lines.

The information regarding ChrPic genotoxicity is controversial. Data from studies with CHO cells have demonstrated that ChrPic may increase the metal’s genotoxicity compared to chromic chloride [64]. On the other hand, Anderson et al. [65] demonstrated that this compound does not induce genotoxicity in human lymphocytes in cell culture, nor in lymphocytes from mice that were supplemented with ChrPic (3 mg·g^−1^ of animal). Therefore, there is conflicting evidence regarding the genotoxicity of ChrPic. There is no data on the genotoxicity of SPGb. However, polysaccharides are known to have low or no genotoxicity. Despite this, since B5 is a new compound, it was necessary to evaluate whether it presented genotoxicity, since this is a parameter required by regulatory agencies such as the OECD for access materials’ biocompatibility when biomedical applications are envisioned [66].

To evaluate the safety of the B5 blend, initially, the genotoxicity of blend 5 was studied by cytokinesis-block micronucleus (CBMN) assay, which is a standardized method that allows us to detect nuclear alteration in binucleated cells such as Micronuclei (MN). MN are originated from whole chromosomes or chromosome fragments that fail to segregate during mitosis [66,67]. The CBMN assay also allows us to evaluate other biomarkers, such as nucleoplasmic bridges (NPBs) and nuclear buds (NBUDs) that are characteristic of chromosomal instability. The CBMN assay was conducted using three different concentrations (0.1, 0.2, and 0.4 mg·mL^−1^) in CHO-k1 cells, which is an established cell line for CBMN assays, and the data are presented in Table 3. It can be observed that regardless of the concentration, the data obtained with the cells exposed to B5 are not significantly different from those obtained with the negative control (NC), which indicates that B5, under the evaluated conditions, does not induced nuclear alterations, and therefore, it is not genotoxic. In addition, the nuclear division index (NDI) demonstrated that the sample did not cause changes in the cell proliferation rate.

One limitation of this evaluation was that B5 was dissolved in DMSO (2%), and therefore, it could not be evaluated at higher concentrations, as those concentrations would reach levels where DMSO itself could present genotoxicity, leading to a false positive. Ethanol can be considered as a substitute for DMSO. However, when the solubility of ChrPic in this solvent was evaluated, it was observed that it was low. Alternatives to replace DMSO are being considered, and it is hoped that soon, a more satisfactory substitute will be found. Propylene glycol and glycerin can serve as alternatives to DMSO. These compounds have been approved by the USFDA and are commonly used in the food and medical industries [68]. Polyethylene glycols (PEGs), especially low-molecular-weight variants, can be considered as a substitute for DMSO. PEG is a compound approved by the USFDA, and its use is popular due to its adjustable properties and well-established safety profile [69]. It is intended to evaluate the solubility of B5 in these solvents in the future.

Given the good results of B5 in cytotoxicity and genotoxicity tests, this compound was evaluated as an antioxidant agent in cells.

### 2.5. Effect of B5 on Hydrogen Peroxide-Induced Oxidative Stress in 3T3 Cells

Oxidative stress occurs continuously, which means that at some point in their life, an individual has likely already suffered from some oxidative damage and may have sequelae caused by it. Therefore, a repairing agent would be indicated for this situation. Additionally, it can be deduced that occasionally oxidative stress reoccurs after the initial damage, in which case the use of an antioxidant that acts concomitantly with the stressor agent and inhibits it is indicated. Finally, the use of antioxidants capable of acting preventively against the damage caused by oxidative stress is highly effective. With this in mind, we decided to evaluate the effect of B5 on three stress conditions with cells that could simulate the situations mentioned above.

**Figure 8:**
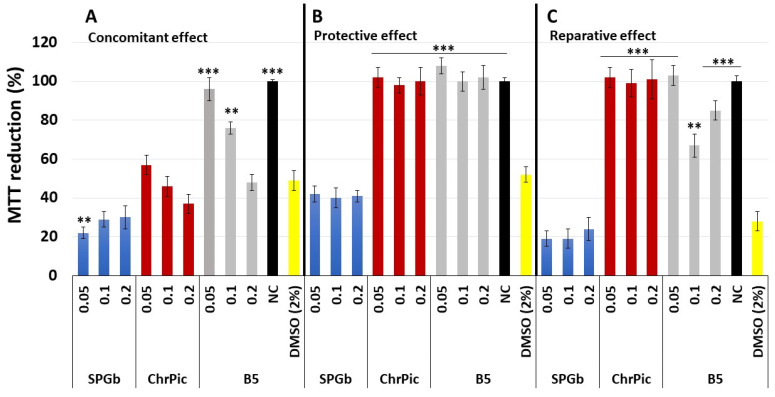
The 3-(4,5-dimethylthiazol-2-yl)-2,5-diphenyl-tetrazolium bromide colorimetric (MTT)-reducing activity of 3T3 cell treated with H_2_O_2_ and samples at different conditions. (**A**) Concomitant effect assay. Cells were incubated with H_2_O_2_ (0.6 mM) along with samples at different concentrations. (**B**) Protective effect assay. Cells were first treated with samples (24 h). Afterwards, the samples were removed, and these cells were exposed to H_2_O_2_ (0.6 mM) for 1 h. (**C**) Reparative effect assay. Cells were treated with H_2_O_2_ (1 h) and subsequently with samples for 24 h. Data are expressed as mean ± standard deviation. ** sample versus DMSO 2% (*p* < 0.05); *** sample versus DMSO (*p* < 0.001). Three independent assays were performed in triplicate, and data were analyzed using ANOVA and Tukey test.

The MTT reduction capacities of 3T3 cells treated with samples (SPGb, ChrPic, and B5) in combination with 0.6 mM hydrogen peroxide (H_2_O_2_) are illustrated in Figure 8A. In this figure, it can be observed that the cells exposed to H_2_O_2_ had their MTT reduction capacity decreased by about 50% compared to the negative control (cells not exposed to peroxide or samples). The presence of SPGb, regardless of the concentration evaluated, was unable to reverse this situation. On the contrary, it further decreased the cells’ ability to reduce MTT to around 30%. The presence of ChrPic also negatively affected the cells’ ability to reduce MTT after exposure to peroxide, but the data were significant only at the highest concentration evaluated (0.1 mg·mL^−1^). In this case, the reduction of MTT by the cells was 37%.

The best results were obtained when using B5. It can be observed that even at the lowest concentration used, there was a significantly similar reduction in MTT of almost 100%. That is, the blend protected the cells from the damage caused by peroxide when they were exposed to cells at the same time. However, as the concentration of blend used increased, this protective property decreased.

In Figure 8B, the results of the 3T3 cell’s capacity to reduce MTT when treated with samples for 24 h and subsequently treated with H_2_O_2_ for 1 h and finally maintained in serum-containing medium for another 24 h (protective effect) are shown.

It can be observed that, as seen in the concomitant assay, the presence of peroxide also significantly decreased the cells’ ability to reduce MTT (~50%). This capacity was further reduced when the cells were exposed to SPGb. On the other hand, both ChrPic and B5 exposure protected the cells from the damage caused by peroxide, as they showed 100% MTT reduction capacity.

In Figure 8C, the results of the 3T3 cell’s capacity to reduce MTT when treated with H_2_O_2_ for 1 h followed by removal of the culture medium containing H_2_O_2_ and the addition of culture medium-containing samples with subsequent treatment for 24 h (repair assay) are shown. The idea here was to determine whether the presence of samples can stimulate the regeneration of damaged cells.

It can be observed that H_2_O_2_ was more aggressive than in previous tests, and the cells’ ability to reduce MTT was around 28%. These values did not improve with exposure to SPGb, regardless of the concentration evaluated. On the other hand, both ChrPic (all evaluated concentrations) and B5 (0.05 mg·mL^−1^) caused the cells to have a percentage of MTT reduction of 100%. B5, with the two highest evaluated concentrations, also induced cells to significantly reduce MTT more than cells exposed to peroxide, but these values were between 60 and 90%.

The absence of repairing activity from SPGb could be due to two possibilities: the first is that the samples have a repairing effect at concentrations above or below those tested, and the second is that the intense cell damage caused by H_2_O_2_ during 1 h of incubation may have been too severe for the antioxidant effects of SPGb to reverse the signaling cascade for cell death. Therefore, the treatments had no effect on cell survival compared to the positive control. This explanation could also be attributed to the activity observed with ChrPic and with B5 (0.05 mg·mL^−1^), which shows that it was possible to transfer the activity from ChrPic to B5. However, no plausible explanation was found for the fact that B5 at higher concentrations (0.1 and 0.2 mg·mL^−1^) was not as efficient in this test as it was at lower concentrations.

In the protective effect assay, it was observed that both ChrPic and B5, under the evaluated conditions, were able to protect cells. However, in this case, it was difficult to evaluate whether the effect observed with B5 was solely derived from ChrPic or whether it also derived from SPGb.

In the concomitant effect assay, B5 (0.01 mg·mL^−1^) was the only condition in which the data caused by H_2_O_2_ were completely avoided. This is interesting because neither SPGb nor ChrPic, when isolated, showed this activity. This indicates that the combined effect of the blend components that was expected to be observed in vitro could only be observed in studies with cells. Here, we also could not find an explanation for why the activity of B5 decreased with increasing concentration. In the future, we intend to create a curve with more data points to identify the limit range for obtaining the desired concomitant effect.

Returning to the context of oxidative damage suffered by the cell population, it was observed that B5 (0.05 mg·mL^−1^) had an excellent antioxidant effect in all three tests, which indicates that it could be used in humans/animals to prevent or combat oxidative stress or even help in the recovery of damage caused by stress. Many compounds, when administered in humans, tend to be quickly metabolized, and their concentration in body fluids decreases, leading to a decrease in their effectiveness. In the case of B5, this may not occur, as it is more effective at lower concentrations. Therefore, its metabolism could be a positive factor for its antioxidant action in vivo.

## 3. Materials and Methods

### 3.1. Materials

Iron (II) sulfate, potassium ferricianyde (III), and sulfuric acid were from Merck (Darmstadt, Germany). Moreover, ammonium molybdate, ascorbic acid, Folin–Ciocalteu reagent, hydrogen peroxide, methionine, nitro blue tetrazolium (NBT), riboflavin, sodium acetate, 3-[4,5-Dimethyl-thiazol-2-yl]-2,5-diphenyltetrazolium bromide (MTT), and sodium phosphate were obtained from Sigma-Aldrich Co. (St. Louis, MO, USA). Other reagents used in this study were of analytical grade.

### 3.2. Extraction of Agaran from G. birdiae (SPGb)

In October 2021, mature seaweeds measuring 35–40 cm in length were collected from Rio do Fogo Beach (Rio Grande do Norte, Brazil—5°16′16″ S/35°22′54″ W) by local fishermen. Prior to laboratory analysis at the Department of Biochemistry, Natural Polymer Biotechnology Laboratory, Federal University of Rio Grande do Norte, RN, the collected seaweeds underwent thorough cleaning to remove residues, encrusted organisms, and epiphytes. Morphological characteristics were used to identify the seaweed [70]. The seaweed displays an upright thallus, reaching a height of up to 40 cm and a width of up to 2.3 mm. Its color ranges from light red to dark red. The thallus is cylindrical in shape, typically branching into two or occasionally four orders. Branching occurs in a subdichotomous or unilateral pattern, with a single main axis or multiple axes originating from a discoid holdfast. The specimens exhibited noticeable cystocarps, measuring approximately 0.9 to 1.2 mm in diameter and around 1.0 mm in height, scattered throughout the thallus. Collection of the materials was carried out with authorization from the Brazilian National System of Management of Genetic Heritage and Associated Traditional Knowledge, SISGEN n° A72AD2B.

The dried seaweed was subsequently ground into a powder and subjected to treatment with twice its volume of ethanol (99.5%, Sigma-Aldrich Co., St. Louis, MO, USA) overnight, following a previously reported method [11]. This procedure aimed to reduce the presence of pigments in the sample. Afterward, the supernatant was discarded, and the resulting powder was dried at 50 °C under adequate ventilation. The desiccated material was then carefully sealed in polyethylene bags and stored at room temperature in a dark environment.

The extraction of SPGb was conducted with modifications based on a previously described method [11]. Approximately 50 g of powdered seaweed were suspended in 500 mL of 0.25 M NaCl, and the pH was adjusted to 8.0 using NaOH. Therefore, this material was submitted to sonication (30 min/60 °C/60 W). Subsequently, 75 mg of Prolav 750 alkaline protease mixture (Prozyn Biosolutions, São Paulo, SP, Brazil) was added for proteolytic digestion. The solution was incubated at 60 °C with agitation and periodic pH adjustments for 24 h. Afterward, the mixture was filtered through cheesecloth to remove solid particles, and the resulting soluble SPGB was precipitated by adding two volumes of ice-cold methanol. The precipitated SPGB was collected by centrifugation (10,000× *g*, 20 min), followed by vacuum drying. The dried SPGB was then re-suspended in distilled water and subjected to analysis.

### 3.3. Chemical Analysis and Monosaccharide Composition

Sulfate content was measured after acid hydrolysis (HCl 6 N, 6 h, 100 °C) using the turbidimetric method described earlier [70]. Protein content was quantified with Coomassie Brilliant Blue reagent and bovine serum albumin as standard, as described in Reference [71]. Phenolic compounds were measured by the Folin–Ciocalteau reagent, as described in Reference [72].

To determine the most effective acid hydrolysis condition for polysaccharides using HCl wherein polymer degradation occurs without destroying the released monosaccharides, SPGb was subjected to hydrolysis with HCl at concentrations of 0.5 M, 1 M, 2 M, and 4 M for 30 min, 1 h, 2 h, and 4 h, respectively. The hydrolysis was carried out at a temperature of 100 °C for all conditions. Subsequently, the material was neutralized, dried, and re-suspended in distillated water, and the content of reducing sugars was determined using the Somogyi–Nelson method [13]. The optimal hydrolysis condition was found to be 2 M HCl for 2 h. Consequently, SPGB was hydrolyzed using these conditions (2 M HCl, 100 °C, 2 h), and the sugar composition was analyzed using a VWR-Hitachi LaChrom Elite^®^ HPLC system equipped with a refractive index detector (RI detector model L-2490). A LichroCART^®^ 250-4 column (250 mm × 4 mm) packed with Lichrospher^®^ 100 NH2 (5 µm) was employed in the system. The column was eluted with a mixture of acetonitrile (gradient-grade for liquid chromatography, Merck, Darmstadt, Germany) and ultrapure water (80:20 *v*/*v*). The sample mass used was 0.2 mg, and the analysis time was 25 min. The following sugars were analyzed as references: arabinose, fructose, fucose, galactose, glucose, glucosamine, glucuronic acid, mannose, and xylose.

### 3.4. Nuclear Magnetic Resonance (NMR) Spectroscopy

Nuclear Magnetic Resonance (NMR) Spectroscopy analyses were performed by dissolving the samples (20 mg) in 600 μL of deuterium oxide (D_2_O). All the NMR analyses were obtained in a Bruker Avance III 600 MHz spectrometer (Bruker BioSpin Corporation, 138 Billerica, MA, USA) equipped with a 5 mm inverse quadruple resonance probe (QXI) at 70 °C. ^1^H-NMR spectra were obtained using number of scans (NS) = 8, spectral width (SWH) of 6393.862 Hz, and size of fid (TD) = 64 k. The 2D-NMR (^1^H/^13^C) HSQCed (Edited Heteronuclear Single Quantum Coherence) analyses were performed using Bruker’s hsqcedetgpsisp2.3 pulse sequence with NS = 32, number of dummy scans (DS) = 128, TD = 2048 (F2) × 200 (F1), SWH = 6393.862 Hz (F2) × 30,182.674 Hz (F1), and relaxation delay = 1.0 s. The chemical shifts were expressed in δ relative to sodium trimethylsilyl propionate (TMSP) at δ = 0.00 in accordance with IUPAC recommendations.

### 3.5. In Vitro Antioxidant Tests

Five in vitro antioxidant tests were used: superoxide radical scavenging assay, hydroxyl radical scavenging assay, ferrous chelating assay, cupric chelating assay, and total antioxidant capacity (TAC). All of them were carried out as described by Presa et al. [63]. SPGB was diluted in distilled water, and ChrPic was diluted in DMSO (100%), both at a concentration of 10 mg·mL^−1^. These solutions were used as stock solutions for the tests.

#### 3.5.1. Determination of Total Antioxidant Capacity (TAC)

Briefly, the solution (1.0 mL) containing the samples (0.1 mg·mL^−1^), ammonium molybdate (4.0 mM), sodium phosphate (28.0 mM), and sulfuric acid (0.6 M) were added into a tube, stirred, and incubated (100 °C, 90 min). The tubes were cooled and were read at 695 nm wavelength. The standard used was ascorbic acid (AA), and the results were expressed as AA equivalent per gram of sample.

#### 3.5.2. Superoxide Radical-Scavenging Assay

As described by Presa et al. [73], 1.0 mL of the samples (at different concentrations) were mixed with 100.0 mM ethylenediaminetetraacetic acid (EDTA), 50.0 mM phosphate buffer (pH 7.8), 13.0 mM methionine, 75.0 mM nitroblue tetrazolium (NBT), and 2.0 mM riboflavin to form a 3.0 mL solution. The entire reaction assembly was carried out in the dark. After 10 min, the formazan forms were monitored at 560 nm. Identical tubes with distilled water and reaction mixture were used as a blank. Gallic acid was used as standard (from 0.01 to 0.6 mg·mL^−1^). The results were expressed according to the equation

% of activity=([Acontrol − Asample]/[Acontrol − Ablank]) × 100

where Acontrol: absorbance of the control tube, Asample: absorbance of the sample tube, and Ablank: absorbance of the blank tube.

#### 3.5.3. Hydroxyl Radical Scavenging Assay

The OH radical scavenging activity of samples was investigated using Fenton’s reaction (Fe^2+^ + H_2_O_2_ → Fe^3+^ + OH^−^ + OH). The data were expressed as the inhibition rate. For OH production, the samples (at different concentrations) were added to 3 mL sodium phosphate buffer (150.0 mM, pH 7.4), which contained 10 mM FeSO_4_·7H_2_O, 10 mM EDTA, 2 mM sodium salicylate, 30% H_2_O_2_. In the control, sodium phosphate buffer replaced H_2_O_2_. After 37 °C for 1 h, OH radical was detected by monitoring absorbance at 510 nm using a microplate reader. Gallic acid was used as a positive control.

#### 3.5.4. Iron-Chelating Assay

The samples at different concentrations were added to a solution containing FeCl_2_ (2.0 mM). After ferrozine (5.0 mM) was added to the mixture, the solution was mixed and kept for 10 min at 37 °C. Ultrapure water was used as blank, and EDTA was used as standard. The samples were monitored at 562 nm using a microplate reader. The results are expressed in accordance with the equation

% of chelation=([Acontrol − Asample]/Acontrol) × 100

where Acontrol: absorbance of the control tube, and Asample: absorbance of the sample tube.

#### 3.5.5. Copper-Chelating Assay

The test was performed in 96-well microplates with a reaction mixture containing different concentrations of samples (0.1–2.0 mg·mL^−1^), copper II sulfate pentahydrate (50 mg·mL^−1^), and pyrocatechol violet (4.0 mM). All wells were homogenized with the aid of a micropipette, and the solution absorbance was measured at 632 nm. The ability of the samples in chelating the copper ion was calculated using the following equation:

(Absorbance of blank) − (Absorbance of the sample)/(Absorbance of the blank) × 100

### 3.6. Production of Blends

Based on the results of the in vitro tests, it was observed that the antioxidant activity of both SPGb and ChrPic reached their maximum when they were at a concentration of 1.0 mg·mL^−1^. Additionally, the lowest concentration of these compounds that was evaluated was 0.5 mg·mL^−1^. Therefore, these two limits (0.5 and 1.0 mg·mL^−1^) were chosen to create the blends, along with selecting an intermediate value between them (0.75 mg·mL^−1^). Thus, these concentrations were combined to form each blend with different ratios of SPGb and ChrPic (Table 4).

### 3.7. 3-[4,5-Dimethyl-thiazol-2-yl]-2,5-diphenyltetrazolium Bromide (MTT) Colorimetric Tests—Cytotoxicity Test

The murine fibroblast (3T3) or Chinese hamster ovary (CHO-K1) cell lines were seeded onto 96-well plates (5 × 10^3^ cells per well) in Dulbecco’s Modified Eagle’s medium (DMEM) supplemented with 10% of fetal bovine serum (FBS) and antibiotics (10,000 U·mL^−1^ penicillin G and 25.0 mg·mL^−1^ streptomycin) and incubated for 24 h at 37 °C and 5% CO_2_. Subsequently, the serum-containing culture medium was removed and replaced with a serum-free medium for starvation. The cells were incubated under the same conditions for another 24 h. After that, the serum-containing medium with the test samples, at concentrations of 0.05 mg·mL^−1^, 0.1 mg·mL^−1^, and 0.2 mg·mL^−1^, were added. DMSO was used as a negative control for ChrPic and B5 at concentrations of 2%, along with a negative control (FBS). The cells were then incubated for 24 h under the same conditions. At the end of the incubation period, the medium was removed, and MTT (1.0 mg·mL^−1^) was added and incubated for 4 h. Afterwards, the MTT was removed, and ethyl alcohol was added under agitation for 15 min at room temperature. The absorbance was measured using a spectrophotometer at 570 nm. Cell cytotoxicity was determined by the MTT colorimetric assay, where % reduction of MTT = absorbance of the test sample/absorbance of the negative control.

### 3.8. Evaluation of Antioxidant Activities of Samples in Cell Culture

#### 3.8.1. Determination of Injury Condition

This method was adapted from Shi et al. [74] and was carried out as follows: To perform this experiment, it was initially necessary to determine the best condition for injury. The chosen cellular model was the murine fibroblast cell line (3T3). Cells were seeded in 96-well plates at a density of 5 × 10^3^ cells/well and allowed to adhere for 24 h at 37 °C and 5% CO_2_. Subsequently, the cells were starved for 24 h. After this period, the medium was replaced with a new medium that did not contain FBS but had different concentrations of H_2_O_2_ (0.1 to 3.0 mM) and was left in contact with the cells for 1 h. Later, the hydrogen peroxide-containing medium was removed and replaced with DMEM culture medium with FBS. After 24 h of incubation, it was observed that the cells’ ability to reduce MTT was decreased by 50% in the condition of 0.6 mM hydrogen peroxide-induced damage. Concentrations greater than 0.6 mM caused damage much greater than 50%, whereas the control without H_2_O_2_ did not show any damage by MTT reduction assay [75].

In the assays below, all treatments had 4 × 10^3^ cells/well that were grown in a 96-well plate with DMEM medium with FBS for 24 h. Subsequently, the cell cycle was synchronized by removing the serum-containing medium and adding serum-free medium and incubating for another 24 h.

#### 3.8.2. Concomitant Test Effect

The serum-free medium was replaced with DMEM containing serum and the samples at different concentrations (0.05 mg·mL^−1^, 0.1 mg·mL^−1^, and 0.2 mg·mL^−1^) plus 0.6 mM H_2_O_2_ and incubated for 1 h. Subsequently, the medium was removed and replaced with serum-containing medium and incubated for 24 h. After incubation, signs of cytotoxicity were determined by the colorimetric assay of 3-(4,5-dimethylthiazol-2-yl)-2,5-diphenyl-tetrazolium bromide (MTT) as described above.

For the H_2_O_2_ control, only DMEM with serum and peroxide were used. For the control without peroxide, DMEM with serum without peroxide was used. DMSO was used as the negative control for ChrPic and B5 at concentrations of 2%.

#### 3.8.3. Protective Test Effect

The serum-free medium was replaced with DMEM with serum containing the samples at different concentrations (0.05 mg·mL^−1^, 0.1 mg·mL^−1^, and 0.2 mg·mL^−1^) and incubated for 24 h. Subsequently, the medium with serum and samples was removed and replaced with serum-free medium with 0.6 mM H_2_O_2_ and incubated for one hour. After this, the medium was removed and replaced with medium with serum. After incubating for 24 h, cell viability was determined by the colorimetric assay of 3-(4,5-dimethylthiazol-2-yl)-2,5-diphenyl-tetrazolium bromide as described above.

DMEM with serum and peroxide was used for the peroxide control. DMEM without serum without peroxide was used for the no peroxide control. DMSO was used as the negative control for ChrPic and B5 at concentrations of 2%.

#### 3.8.4. Reparative Test Effect

For this assay, the serum-free medium was changed to serum-free DMEM with 0.6 mM H_2_O_2_ and incubated for one hour. The cells in the control without peroxide were treated with serum-containing medium without hydrogen peroxide. After one hour of exposure to hydrogen peroxide, the medium was removed from the plate and replaced with serum-containing medium with samples at different concentrations (0.05 mg·mL^−1^, 0.1 mg·mL^−1^, and 0.2 mg·mL^−1^). After incubating for 24 h, signs of cytotoxicity were determined by the colorimetric assay of 3-[4,5-dimethylthiazol-2-yl]-2,5-diphenyl-tetrazolium bromide by replacing the supernatant with serum-free medium containing 1 mg·mL^−1^ MTT and incubating for 4 h at 37 °C. After this incubation period, the supernatant was removed, and the formazan crystals were dissolved in 99% ethanol. The plate was agitated for 15 min at room temperature for homogenization, and absorbance was measured at 570 nm in a microplate reader.

A control with peroxide was also performed using serum containing DMEM and peroxide. For the control without peroxide, serum containing DMEM without peroxide was used. The absorbance of the control without peroxide was considered as 100% reduction of MTT, and the values of treated cells were calculated as a percentage of the negative control.

### 3.9. In Vitro Evaluation of the Genotoxicity by the Cytokinesis Blocking Micronucleus Test (CBMN)

CHO-K1 cells were cultivated in 24-well plates at a density of 2 × 10^4^ cells/well in 500.0 μL of D-MEM medium supplemented with 10% FBS, 1% antibiotics and 1% glutamine. They were kept at 37 °C in 5% CO_2_. After 24 h, the culture medium was removed from the wells, and 500 μL of medium containing the samples to be analyzed were added to each well, which were B5 at concentrations of 0.1; 0.2 and 0.4 mg·mL^−1^, plus positive (1.0 μg·mL^−1^ Mitomycin C) and negative (DMEM) controls (all in triplicates). After 24 h, the medium was removed and the cells were washed with 400 μL of PBS, and 500 μL of complete medium containing Cytochalasin B at 5 μg·mL^−1^ (CAS: 14930-96-2; Sigma-Aldrich Co., St. Louis, MO, USA) was added, which acts as a cytokinesis blocker. In the wells of the positive control, Mitomycin C (1 μg·mL^−1^) was also added.

After 24 h, the medium was removed, and the cells were washed with 400 μL of PBS. Subsequently, 200 μL of trypsin (0.025%) were added for 5 min and inactivated with 600 μL of medium. The released cells were transferred to 15 mL conical tubes, centrifuged at 1200 rpm for 8 min, and the supernatant was discarded.

Then, the cells were resuspended in 3 mL of chilled fixative solution (1/9 acetic acid/methanol) slowly and gently to avoid breaking the cell membrane, and the material was centrifuged at 1200 rpm for 8 min. The supernatant was discarded, and cells were resuspended in 400 μL of fixative solution. Then, cells were homogenized slowly, and 60 μL of this suspension was dripped onto clean and moist glass slides that were under a water bath (60 °C).

The slides were stained for 5 min with 5% Giemsa, washed with distilled water, and 3000 binucleated cells [67] were counted at a magnification of 400×. Of these 3000 cells, the first 500 cells (with intact cytoplasm and well-defined nuclei) were counted for the determination of the cell division index (CNDI) using the formula NDI = [M1 + 2 (M2) + 3 (M3) + (M4)/Total number of cells], where M1 is the number of mononuclear cells, M2 is binuclear, M3 is trinuclear, and M4 is tetranuclear cells.

A result close to two indicates a regular cell cycle, while lower results indicate a delay in the cell cycle. NT corresponds to 500 cells. After the determination of the nuclear division index (NDI), binucleated cells were quantified for the presence of micronuclei (MN), nucleoplasmic bridges (NBs), and nuclear buds (NBUDs) that determine nuclear alteration. The selection criteria, as well as the counting of binucleated cells with MN, NB, and NBUD presence, were defined according to the OECD TG 487 guideline [66].

### 3.10. Statistical Analysis

The data obtained were expressed as mean ± standard deviation (N = 3) in triplicates using Past^®^ 6.0 software after testing for normality using Shapiro Wilk test and were confirmed to be paired (*p* > 0.05). Differences between samples were verified by one-way ANOVA followed by Tukey’s pairwise test (*p* < 0.05) to identify which tested samples showed significant differences.

## 4. Conclusions

A sulfated agaran (SPGb) was purified from the seaweed *G. birdiae*, and its identity was confirmed by NMR. SPGb showed excellent activity as a chelator for both copper and iron ions but did not exhibit activity in the hydroxyl- or superoxide ion-scavenging tests. On the other hand, ChrPic showed approximately 100% activity in these two tests. However, ChrPic exhibited low copper chelating activity and no iron chelating activity. SPGb did not show cytotoxicity, whereas the cytotoxic effect of ChrPic was observed starting at 0.2 × 5 mg·mL^−1^.

It was possible to obtain five blends composed of SPGb and ChrPic. These showed in vitro antioxidant activity with different degrees of action. The presence of SPGb at the same concentration as ChrPic, 1.0 mg·mL^−1^ (B4 blend), clearly reduces the hydroxyl and superoxide scavenging activity of ChrPic. This effect is observed within a specific concentration range, as evidenced by B5, which contains 0.75 × 5 mg·mL^−1^ of each compound and exhibits 100% activity in both tests. However, we were unable to find a justification for the observed result with B4. Nevertheless, these findings demonstrate that there are concentration ranges where one compound from the blend can interfere with the action of another compound, leading to a reduction in the overall activity of the blend. Among the blends, B5 stood out the most. This blend was able to reduce the cytotoxicity observed with ChrPic and still presented activity similar to ChrPic in most of the tests, except in the test that exposed the cells to peroxide concomitantly with B5, when the activity of B5 was 3 times higher than that of ChrPic. This shows the importance of the presence of SPGb in the composition of the blend. In addition, B5 did not present genotoxic effect. These data indicate B5 as a product to be used in future in vivo tests to confirm its antioxidant action.

ChrPic is a compound known for its antioxidant activity and various other properties, as described in the introduction. However, caution is advised when using ChrPic due to its cytotoxic effects, as indicated by both in vitro and in vivo data. Here, the blend B5 was developed, which exhibited antioxidant properties in multiple tests. Interestingly, B5 demonstrated superior activity compared to ChrPic in certain assays, and it was able to eliminate the cytotoxicity associated with ChrPic. Further in vitro and in vivo experiments are required to assess whether B5 retains the additional properties of ChrPic and SPGb. Such findings would indicate that B5 is not only an effective antioxidant agent, but also a promising nutraceutical with immunomodulatory properties, capable of addressing various diseases and metabolic disorders.

## Figures and Tables

**Table 1:** Chemical composition of SPGb.

Sample	Sugar (%)	Sulfate (%)	Protein (%)	Phenolic (%)	Molar Ratio			
					Gal	Glc	Ara	Xyl
SPGb	79.1 ± 0.4	10.2 ± 0.6	0.6 ± 0.1	nd	1.0	0.3	0.2	0.1

Gal—galactose; Xyl—xylose; Ara—arabinose; Glc—glucose. nd—not detectable.

**Table 2:** Chemical shift assignments of the NMR spectra of SPGb based on 2D-NMR experiment.

Unit	Structural Unit	Chemical Shifts, δ (ppm) ^a^					
		H1	H2	H3	H4	H5	H6
		C1	C2	C3	C4	C5	C6
A	α-L-galactose-6-sulfate (L-6S)	5.31	3.85	4.00	4.28	nd	4.30
		101.7	69.8	69.3	78.2	71.0	67.7
B	3,6-α-L-anhydrogalactose	5.12	4.10	4.52	4.65	4.55	4.11
		98.7	70.6	80.8	77.2	75.3	69.4
C	β-D-Galactose	4.54	3.66	3.75	4.15	3.75	3.80
		102.4	70.8	82.4	68.8	75.8	61.4
D	β-D-Galactose linked to L-6S units	4.41	3.76	nd	nd	nd	nd
		103.8	70.8	nd	nd	nd	nd

^a^ Chemical shifts are relative to the internal standard, trimethylsilyl propionic acid (δ = 0 ppm).

**Table 3:** Analyses of nuclear alterations and the nuclear division index (NDI) on CHO-k1 cells exposed to of B5, by CBMN test.

Samples	MN	NPBs	NBUDs	NDI
NC	6.0 ± 1.8	4.3 ± 0.9	5.8 ± 0.8	2.0 ± 0.0
PC	15.3 ± 0.6 *	11.0 ± 0.6 *	13.7 ± 2.2 *	2.0 ± 0.0
B5 0.1 mg·mL^−1^	7.1 ± 1.0	3.6 ± 0.2	4.6 ± 1.0	1.9 ± 0.0
B5 0.2 mg·mL^−1^	7.0 ± 1.2	3.3 ± 1.0	4.6 ± 1.6	1.9 ± 0.0
B5 0.4 mg·mL^−1^	8.3 ± 1.1	3.2 ± 1.3	3.1 ± 0.8	1.9 ± 0.0

CHO-k1 cells maintained in the growth medium were used as negative (NC), and cells in the growth medium containing mitomycin C were used as a positive control (PC). The frequencies of nuclear alterations in Micronuclei (MN), Nucleoplasmic bridges (NPBs), and Nuclear buds (NBUDs) were determined in 3000 binucleate cells and presented as the number of occurrences per 1000 binucleated cells. Three independent assays were performed in triplicate, and data were analyzed using ANOVA followed by Dunnett’s test. Values were expressed as the mean ± standard deviation, and * *p* < 0.005 was considered to be statistically significant.

**Table 4:** Composition of different blends.

Blend	ChrPic	SPGb
B1	0.5 mg·mL^−1^	0.5 mg·mL^−1^
B2	1.0 mg·mL^−1^	0.5 mg·mL^−1^
B3	0.5 mg·mL^−1^	1.0 mg·mL^−1^
B4	1.0 mg·mL^−1^	1.0 mg·mL^−1^
B5	0.75 mg·mL^−1^	0.75 mg·mL^−1^

## Data Availability

The data presented in this study are available on request from the corresponding author.
